# Supplementary material for: End Sequence Analysis Toolkit (ESAT) expands the extractable information from single-cell RNA-seq data
Source: Genome Res. 2016 Oct;26(10):1397–410. doi: 10.1101/gr.207902.116 (PMC5052061; doi:10.1101/gr.207902.116)
Supplement: Supplemental Material [file supp_gr.207902.116_Supp_Materials.tar.gz › ESAT-esat_v0.1/doc/html/classbroad_1_1core_1_1math_1_1_fisher_combined_probability_test.html]

ESAT: broad.core.math.FisherCombinedProbabilityTest Class Reference


ESAT


- Main Page
- Related Pages
- Packages
- Classes
- Files

- Class List
- Class Index
- Class Hierarchy
- Class Members

All Classes Namespaces Files Functions Variables Enumerator Pages

- broad
- core
- math
- FisherCombinedProbabilityTest

Public Member Functions |
List of all members

broad.core.math.FisherCombinedProbabilityTest Class Reference

|  |  |
| --- | --- |
| Public Member Functions | |
|  | FisherCombinedProbabilityTest (double[] pvals) |
|  | |
|  | FisherCombinedProbabilityTest (Collection< Double > pvals) |
|  | |
| double | getTestStatistic () |
|  | |
| double | getNullDistDegreesOfFreedom () |
|  | |
| double | getCombinedPvalue () |
|  | |

## Detailed Description

Author
:   prussell Fisher's method to combine results of several independent tests bearing upon the same overall hypothesis

## Constructor & Destructor Documentation

|  |  |  |  |  |  |
| --- | --- | --- | --- | --- | --- |
| broad.core.math.FisherCombinedProbabilityTest.FisherCombinedProbabilityTest | ( | double[] | *pvals* | ) |  |

Instantiate with array of P values

Parameters
:   |  |  |
    | --- | --- |
    | pvals | The P values from the independent tests |

|  |  |  |  |  |  |
| --- | --- | --- | --- | --- | --- |
| broad.core.math.FisherCombinedProbabilityTest.FisherCombinedProbabilityTest | ( | Collection< Double > | *pvals* | ) |  |

Instantiate with collection of P values

Parameters
:   |  |  |
    | --- | --- |
    | pvals | The P values from the independent tests |

## Member Function Documentation

|  |  |  |  |  |
| --- | --- | --- | --- | --- |
| double broad.core.math.FisherCombinedProbabilityTest.getCombinedPvalue | ( |  | ) |  |

Get the P value of the test statistic

Returns
:   The P value with respect to the null chi square distribution

Here is the call graph for this function:

|  |  |  |  |  |
| --- | --- | --- | --- | --- |
| double broad.core.math.FisherCombinedProbabilityTest.getNullDistDegreesOfFreedom | ( |  | ) |  |

Get the number of degrees of freedom of the null chi square distribution

Returns
:   The number of degrees of freedom

Here is the caller graph for this function:

|  |  |  |  |  |
| --- | --- | --- | --- | --- |
| double broad.core.math.FisherCombinedProbabilityTest.getTestStatistic | ( |  | ) |  |

Get the Fisher test statistic X^2

Returns
:   The test statistic

Here is the caller graph for this function:

---

The documentation for this class was generated from the following file:

- java/broad/core/math/FisherCombinedProbabilityTest.java


---

Generated on Fri Aug 15 2014 15:10:52 for ESAT by  

 1.8.7
